# Supplementary material for: Depression, anxiety, burnout and empathy among Spanish medical students
Source: PLoS One. 2021 Dec 2;16(12):e0260359. doi: 10.1371/journal.pone.0260359 (PMC8638989; doi:10.1371/journal.pone.0260359)
Supplement: Attachment — Submitted filename: Rebuttal letter.docx [file pone.0260359.s001.docx]

**REBUTTAL LETTER PONE-D-21-06742**

Murcia, April 11th 2021

Agustín Martínez Molina, Ph.D.
Academic Editor
PLOS ONE

Dear Editor, this a letter to explain all changes made to our original manuscript as submitted originally (PONE-D-21-06742),

There were no reviewers criticisms but only the editor’s comments: “(to add) description of the measures, completing the analyzes and resubmitting your work to begin the review process”.

These two comments have been answered in the new version PONE-D-21-11578, including a new table 2 that shows the bivariate analysis between all 5 variables. In the new manuscript, all these new sentences have been marked in yellow. Some new references were added so the references list has been also modified.

We hope that the editor is satisfied with these data. In any case, we remain at your disposal to include any other information that might be needed.

Yours truly,

Joaquin Garcia-Estan
